# Reproductive Biology and Functional Response of *Dineulophus phtorimaeae*, a Natural Enemy of the Tomato Moth, *Tuta absoluta*

**DOI:** 10.1673/031.012.15301

**Published:** 2012-12-28

**Authors:** Vivina Savino, Carlos E. Coviella, María G. Luna

**Affiliations:** ^1^ Programa de Ecología Terrestre, Departamento de Ciencias Básicas e Instituto de Ecología y Desarrollo Sustentable (INEDES). Universidad Nacional de Luján, Argentina. Rutas 5 y 7, Luján (6700), Argentina; ^2^ Centro de Estudios Parasitológicos y de Vectores (CEPAVE —CCT La Plata- CONICET-UNLP), Calle 2 Nro. 584 (1900) La Plata, Argentina

**Keywords:** host-feeding, ovigeny, parasitoid, type I functional response

## Abstract

The tomato moth, *Tuta absoluta* (Lepidoptera: Gelechiidae), is a major pest in South America and is at present an important invasive species in the Mediterranean Basin. The larval stadium mines leaves, stems, and fruits, and chemical control is the most used control method in both its original range and the invaded distribution regions. Since current *T. absoluta* control strategies seem limited, biological control is a prominent tool to be applied abroad. The naturally occurring larval ectoparasitoid in Argentina and Chile *Dineulophus phtorimaeae* (Hymenoptera: Eulophidae) has been reported to have potential biocontrol efficiency. In this study, the ovigeny strategy of *D. phtorimaeae* was analyzed throughout the adult female lifetime, and the functional response of females offered a range of 2–15 *T. absoluta* larvae was measured over a 48-hour period. Mean *D. phtorimaeae* egg load was 4.15 eggs, and egg production resulted in extremely synovigenic behavior. Meanwhile, a decreasing number of eggs, due to resorption, was found. Proportions of attacked (host-fed and/or parasitized) and only host-fed hosts by the ectoparasitoid were density independent for the tested host range, exhibiting a type I functional response to *T. absoluta*, with an attack rate of 0.20 host larvae. Meanings of this reproductive strategy in evolutionary time as well as the consequences for augmentative biological control programs are discussed.

## Introduction

The moth *Tuta absoluta* (Meyrick) (Lepidoptera: Gelechiidae), a pest of tomato, *Solanum lycopersicum* L. (Solanales: Solanaceae), causes significant economic loss to tomato cropping in many countries in South America (Botto et al. 1999; Siqueira et al. 2000; Magalhaes et al. 2001). Although this is an endemic Neotropical pest, it has acquired a wider geographical distribution after its unintended introduction in other tomato production regions, such as southern Europe and northern Africa, during the last decade (EPPO Bulletin 2005; Urbaneja et al. 2007; Netherlands Plant Protection Service 2009; Speranza et al. 2009; Desneux et al. 2010). In its new regions, *T. absoluta* has spread extremely fast, becoming a potential threat to the world tomato production (Desneux et al. 2011).

*T. absoluta* damage is caused by the feeding activity of larvae. All larval stadia feed on the mesophyll of the leaf and produce mines and galleries that reduce the photosynthetic capacity of the plant. At high density levels, it can affect mature fruits and stems, and indirectly generate an entrance way for pathogens, thus severely degrading crop commercial value.

Several strategies are commonly used to control *T. absoluta* in its original and invaded distribution regions: pest monitoring, agricultural practices, chemical control, management by means of semiochemicals (mating disruption and mass trapping), and to a lesser extent, biological control using *Bacillus thuringiensis*, the egg parasitoid *Trichogramma pretiosum*, and the predators *Nesidiocoris tenuis* and *Podisus nigrispinus* (Miranda et al. 1998; Torres et al. 2002; Garcia et al. 2005; Polack et al. 2002; Mollá et al. 2011). Since *T. absoluta* has a widespread distribution, the current control techniques seem to be limited. Particularly, control by insecticides might be inefficient, as it is complicated by the appearance of resistant populations and, even more importantly, produces adverse effects to human health and to the environment (Lietti et al. 2005; Bielza 2010). As a feasible phytosanitary measure, biological control within an integrated pest management program is a challenging but potentially very beneficial tactic to develop.

Among a list of more than 50 species of *T. absoluta* entomophagous insects reported elsewhere (Garcia Roa 1989; Consoli et al. 1998; Miranda et al. 1998; Faria et al. 2000; Luna et al. 2010, 2011; Desneux et al. 2010; Medeiros et al. 2011), some of them have been pointed out as potential biocontrol agents by means of augmentative releases (Pratissoli et al. 1998; Giustolin et al. 2001; Torres et al. 2002; Luna et al. 2007; Faria et al. 2008; Sanchez et al. 2009; Luna et al. 2010; Mollá et al. 2011). However, efficient control has not been demonstrated yet, with basic biological research still needed if a successful integrated pest management program is to be developed for this pest.

The larval ectoparasitoid *Dineulophus phtorimaeae* de Santis (Hymenoptera: Eulophidae) is a species native to Argentina and Chile and has been the subject of laboratory and field studies in order to gain knowledge on its systematics (de Santis 1983) and basic biological and ecological traits (Vargas 1970; Larrain 1986; Colomo et al. 2002; Luna et al. 2010). As an idiobiont species, it halts host development after attacking it by the injection of venom. Upon eclosion, *D. phtorimaeae* larvae grow protected inside the leaf mines and pupate *in situ*. It is a solitary parasitoid that prefers the third-instar larval host to oviposit. One other positive trait worth mentioning is its apparent specificity for *T. absoluta*.

To continue evaluating *D. phtorimaeae* potential as a biological control agent, its reproductive behavior and its functional response need to be addressed. Luna et al. (2010) found evidence that *D. phtorimaeae* practices non-concurrent destructive host feeding, that is, the adult female consumes host haemolymph and tissues without ovipositing, causing the death of the host (Jervis and Kidd 1986). Among the mechanisms described for this behavior, punctures with the ovipositor and mouthparts and the construction of a feeding tube are mentioned. Occurrence of host feeding in wasp parasitoids is related to ovigeny strategy, which can be pro-ovigenic (all eggs are mature at female's emergence) or synovigenic (the female develops most of its lifetime egg complement after emergence) (Jervis and Copland 1996; Quicke 1997). Jervis et al. (2001) devised an “ovigeny index,” which measures the degree of early-life concentration of egg production. An index of 1 refers to strict pro-ovigeny, whereas an index of 0 indicates extreme synovigeny. For idiobiont parasitoids, these authors pointed out a relationship between longer life expectancy and host-feeding behavior, and linked it to synovigeny and the production of few anhydropic (rich in yolk) eggs. Another reported trait for synovigenic parasitoids is their ability to reabsorb mature eggs, which implies the use of materials in the yolk to fuel somatic maintenance during periods of host or non-host food (floral and extrafloral nectar and honeydew) scarcity. Egg resorption is a mean to reallocate resources (Jervis and Kidd 1986; Bernstein and Jervis 2008). Thus, a non-concurrent, destructive host feeding behavior, as shown by *D. phtorimaeae*, particularly if associated to a synovigenic strategy, could have a significant impact on its ability to act as a successful biocontrol agent.

Another point of interest in parasitoid biology is the response of the ovipositional rate as a function of host density, i.e., the functional response. This trait has been considered as one of the most important aspects in the dynamics of predator-prey interactions, and three main types are described: independency (type I), direct (type III), and inverse density dependency (type II) of parasitoid attack to host density (Holling 1959; Hassell 2000). Particularly for biocontrol programs, functional response studies of potential candidates deal with the estimation of two parameters, the attack rate (a') and the handling time (T*h*), to make comparisons or predictions about possible control success. In a type I functional response, a' is a constant proportion of killed hosts, in a type II it gradually declines, and in a type III it linearly increases at low host densities and decreases at high host densities (Juliano 2001; Fernandez Arhex and Corley 2003; Luna et al. 2007). T*h* is defined as the time needed to manipulate the host and lay the eggs. Many parasitoids kill significant numbers of hosts by feeding, besides parasitizing on them. For parasitoids that practice non-concurrent, destructive host feeding, they can act as predators as well as parasites. Although this behavior could contribute to a decrease in host density, limited studies have been done in the laboratory to understand its relevance to the functional response. In theory, to maximize reproductive success over the female's lifetime, it is predicted that parasitoids should manage their feeding or ovipositing behavior on the basis of the female's nutritional status and host density, which affect their functional responses (Collins et al. 1981; Bai and Mackauer 1990). In search for the best biocontrol agent, most programs assume that a successful attack by a parasitoid results in egg laying. However, this is not clear for many host-feeding species (Kidd and Jervis 1989), although Jervis et al (1996) indicated that destructive host-feeders are probably better biological control agents.

Based upon some known positive *D. phtorimaeae* attributes, the objectives of this study were to elucidate novel aspects of its biology by (1) explaining the type of ovigeny strategy (maturation, egg load, and size) and (2) determining its functional response. This knowledge is relevant when selecting potential biocontrol agents for *T. absoluta* management in its native distribution, and also when assessing an eventual introduction into its invasive range.

## Materials and Methods

### Insect colonies

A *T. absoluta* colony was established from material collected from commercial organic tomato crops located at the La Plata Horticultural Belt (La Plata City, Buenos Aires, Argentina), a major agricultural region in the country. Rearing protocol was described elsewhere (Luna et al. 2007). Briefly, *T. absoluta* larvae were fed with fresh and waterrinsed tomato leaves, extracted from potted tomato plants maintained in an experimental greenhouse at the Centro de Estudios Parasitológicos y de Vectores. Adults were kept in 40 × 40 × 40 cm voile-meshed cages and provided a honey solution (*ad libitum*) and potted tomato plants (three fully expanded leaves) as substrate for oviposition. Cages were checked every two days to replace plants, which were then placed individually in plastic boxes (20 × 20 × 30 cm) to allow for egg hatching and first-instar larvae strolling to construct mines. Thereafter, infested leaves were cut and placed in plastic containers and maintained until pupation.

To set up a *D. phtorimaeae* colony, parasitoid specimens were collected along with those from the host. All mines in damaged leaves were observed using a stereo microscope, using it to search for immobile hosts and ectoparasitoid larvae and pupae. The immobile hosts and ectoparasitoid larvae and pupae were maintained in glass vials (5 mL) until parasitoid adult emergence, sexed, and paired up to allow mating. Adults were provided with separate drops of water and honey solution, but without hosts until the setting of experiments.

Both colonies were maintained in a walk-in room at 25 ± 2° C, 16 hour day-length, and 70 ± 10% relative humidity.

### Determination of ovigeny

In order to detect the type of ovigeny strategy (pro- or synovygenic) of *D. phtorimaeae*, the criteria described by Jervis et al. (2001) were followed by recording lifetime egg production as an indicative of the relative numbers of immature and mature oocytes in the ovaries. Previous work (Luna et al. 2010) indicated that the *D. phtorimaeae* oviposition curve was quite uneven and peaked at the fourth day after mating (five days old). To determine parasitoid ovigeny strategy, mated adult females were exposed to 6–8 hosts (third instar *T. absoluta* larvae) at three, five, and seven days after emergence for 48 hours (n = 10 per age). During the experiments, parasitoids were maintained with honey and water (*ad libitum*). After each trial was completed, hosts were recognized as ‘paralyzed’ (host-fed) or ‘parasitized’ (presence of an external parasitoid egg). Since *D. phtorimaeae* females enter into the mines to attack concealed *T. absoluta* larvae, host feeding could not be directly observed. Instead, the onset of permanent paralysis and yellowish coloration were used to determine that parasitoid behavior (Luna et al. 2010). Wasp females were dissected on a drop of glycerin using a BX51 microscope equipped with a digital camera DP71 (Olympus UK Ltd., http://www.olympus.co.uk) and a DP Manager imaging system software (v. 3.1.1.208, Olympus Corporation, http://www.olympus-global.com/) to record (a) egg load (number of mature oocytes) and (b) oocyte size (length in µm). Ten female wasps were also dissected at emergence (day = 0) in order to count the number of eggs. Evidence of egg resorption was also registered. Descriptions were made based on microphotographs of the dissections.

To assess possible differences in egg maturation over the adult female lifetime, egg load and size produced by females at each age were analyzed by using one-way analysis of variance (ANOVA) with an α = 0.05. The statistical analyses were performed using the software package Statistica (Statsoft 2007). Treatment means were separated using unequal N Tukey honestly significant difference (HSD) test with α = 0.05.

Additionally, possible effects of host deprivation on *D. phtorimaeae* ovipositional behavior throughout the adult female period was studied by recording the number of hosts paralyzed (host-fed) and parasitized at the three ages considered.

### Functional response

To determine *D. phtorimaeae* functional response, an experiment was conducted in which newly emerged adult wasps were paired-up and placed in polystyrene clear containers (500 mL) as mating arenas. Individuals were provided with a honey solution and water (*ad libitum*) and allowed to copulate for 24 hours. Then, males were removed and females were kept for the next four days until they were offered a range of six host densities (2, 3, 4, 6, 8, 10, and 15) for a fixed 48-hour time period. The chosen density range represented the actual *T. absoluta* abundance in the field, being the maximum value two-fold the maximum injury level reported (8 larvae/plant; Bajonero et al. 2008). In this study, third-instar *T. absoluta* larvae already installed in mines were used. Each density treatment was replicated 10 times. At the end of the experiment, the exposed host larvae were removed from the container and checked for symptoms of paralysis (host-fed) and/ or parasitism using a stereoscope.

Since both parasitized and host-fed hosts were paralyzed and died because of the attacks, a decision was made to analyze the overall attack functional response, regardless of whether the attacks led to an ovipositing or a host-feeding event.

In order to determine the shape of the functional response, logistic regressions were performed for the proportion of hosts (a) attacked (N_a_/N_0_), which includes those only host-fed and/or parasitized hosts, and (b) only host-fed (Np/N0), as a function of the number of host densities offered (N_0_), were performed (Juliano 2001; Fernandez Arhex and Corley 2004):
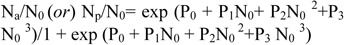

where P_0_, P_1_, P_2_, and P_3_, are the intercept, linear, quadratic, and cubic coefficients, respectively. Estimates of parameters P_0_ to P_3_ were obtained by applying the non-linear estimation module (user-specified regression, least squares) of Statistica (Statsoft 2007) to a dichotomous variable that equals 0 for surviving larvae and 1 for attacked and only paralyzed larvae. Because the cubic expression had redundant predictors, the model was reduced by eliminating that term. By using this method, three different results were expected: 1) if none of the parameters, P_0_, P_1_, P_2_, or only P_0_ is significant, there is a type I functional response; 2) If P_1_ is significantly negative, a type II functional response exists; and 3) if P_1_ and P_2_ are significantly positive and negative, respectively, a type III functional response exists (Frewin et al. 2010; Sueldo et al. 2010).

Once the shape of the functional response was determined, linear regression was performed in order to estimate the parasitoid instantaneous attack coefficient (a'), a conventional parameter associated with Holling-type models (Holling 1966). In this study, the following type I model equation was used:
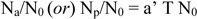

where T is the time that hosts were exposed to the parasitoid. A non-linear regression procedure based on the Levenberg-Marquardt method (Statistica Statsoft 2007) was used to estimate the parameter a'.

## Results

### Determination of ovigeny

**Figure 1.**
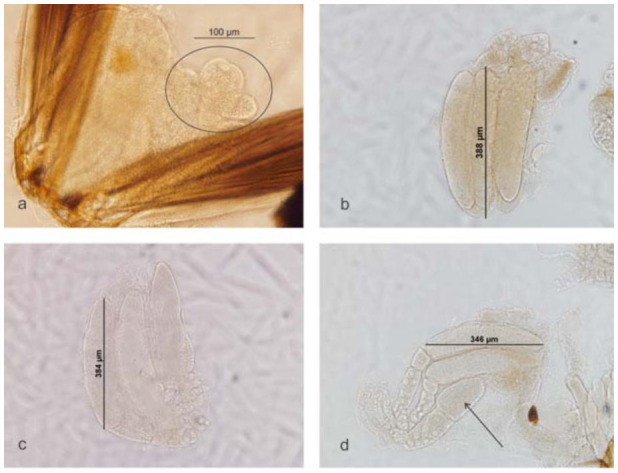
Ovaries dissected from *Dineulophus phtorimaeae* females at different ages: (a) newly emerged; (b) 3 days old; (c) 5 days old; and (d) 7 days old. In (a) the circle shows masses of trophocytes. In (d), the arrow indicates an immature egg forming. High quality figures are available online.

Microscope diagnosis of egg production over the lifetime of female *D. phtorimaeae* showed that naive females (day 0) lack mature oocytes, presenting only immature ones with masses of trophocytes (Figure 1a). Additionally, creamy-white, opaque, hymenopteriform masses of eggs were found at the other three ages tested (Figure 1b, c, d). The total mean egg load for female *D. phtorimaeae* was 4.15 ± 0.4 eggs (mean ± SE, n = 27). A significant decrease in egg load with an increase in adult female age was found (ANOVA *F* = 4.20; df = 2, 24; *p* = 0.03) (Table 1), with an apparent resorption rate of about one egg in 48 hr. Interestingly, 20% of 3-day-old females had no mature eggs, and few immature oocytes were observed being part of mature egg masses in 7-day-olds (Figure 1d).

**Figure 2.**
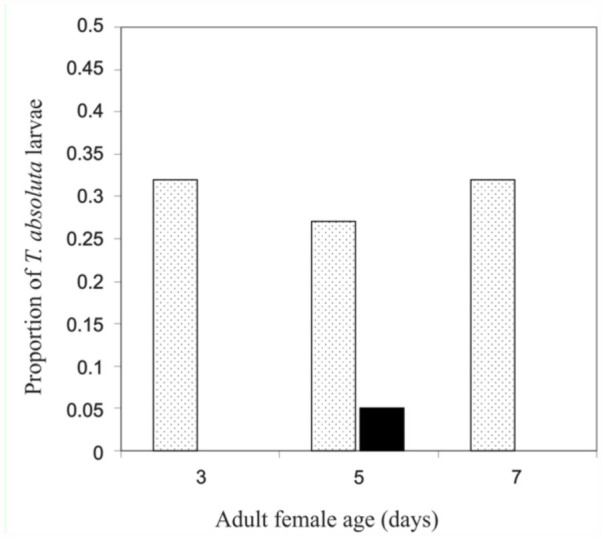
Proportion of paralyzed (dotted bars) and parasitized (bold bar) *Tuta absoluta* larvae by three different ages of *Dineulophus phtorimaeae* adult females (n = 29; mean). High quality figures are available online.

*D. phtorimaeae* females killed around 30% of *T. absoluta* larvae at the three ages considered. Three and 7-day-old wasps only practiced host-feeding without parasitizing; at 5 days old they parasitized 5% of the offered hosts (Figure 2). Paralyzed hosts were observed carefully with a microscope in order to determine a *D. phtorimaeae* host-feeding mechanism, but no noticeable evidence of parasitoid feeding marks or punctures in *T. absoluta* attacked larvae were found.

Eggs averaged a size of 352.79 ± 5.58 µm (mean ± SE, n ;= 26) length. No change in mature egg length over the range of adult female ages studied was found (*F* = 0.29; df = 2, 23; *p* = 0.75) (Table 1).

### Functional response

**Figure 3.**
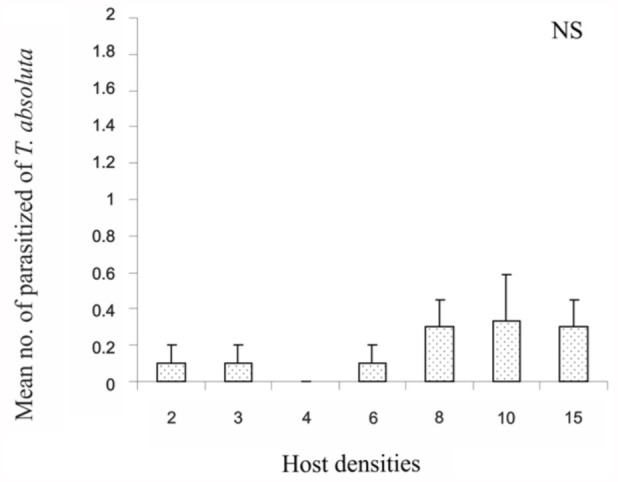
Number of *Tuta absoluta* larvae parasitised by *Dineulophus phtorimaeae* in relation to host density (n = 72; mean ± SE). NS = not significant, *p* > 0.01. Linear regression: y = 0.017 + 0.023x, significant at 95% confidence. High quality figures are available online.

An examination of fecundity yielded, as expected, a low mean number of parasitized *T*. *absoluta* larvae by *D. phtorimaeae* at each host density (Figure 3). It averaged a total of 0.18 parasitized host larvae (± 0.06 SE, n = 72). A slight increase in mean parasitized hosts was observed at higher *T. absoluta* densities, but non-significant linear regression was found (*p* = 0.87). The maximum number of parasitized hosts was 3 at density = 10.

**Table 1.**
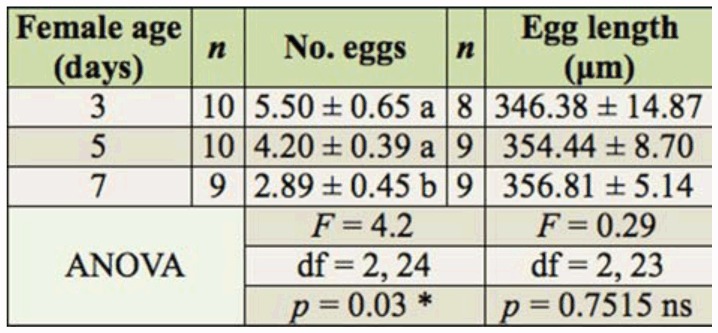
Measurements (mean ± SE) of microscope diagnosis of *Dineulophus phtorimaeae* egg production over the female lifetime. Different letters (a, b) indicate means that are statistically significantly different from each (Tukey's unequal N HDS test; *p* = 0.05, df = 24). ns = not significant. ^*^ = Significant at *p* < 0.05.

**Table 2.**
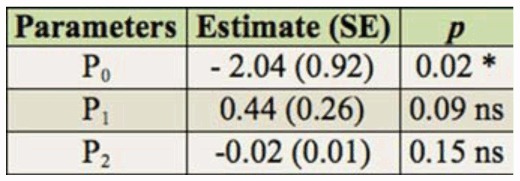
Logistic regression analysis of proportion of attacked (host-fed and/or parasitized) *Tuta absoluta* larvae by *Dineulophus phtorimaeae*. n = 144. ns = not significant. ^*^ = significant at *p* = 0.05.

**Table 3.**
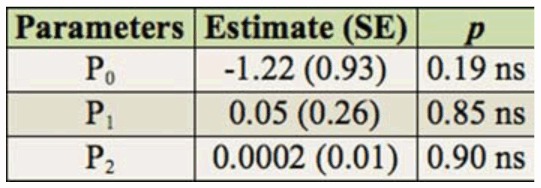
Logistic regression analysis of proportion of paralyzed (host-fed) *Tuta absoluta* larvae by *Dineulophus phtorimaeae*. n = 144. ns = not significant.

**Table 4.**
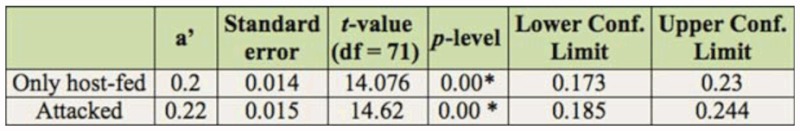
Attack coefficients (a') of the number of attacked (host-fed and/or parasitized), and only host-fed *Tuta absoluta* larvae by *Dineulophus phtorimaeae*, in a 48-hr experimental period. n = 72. ^*^ = significant at α = 0.05.

**Figure 4.**
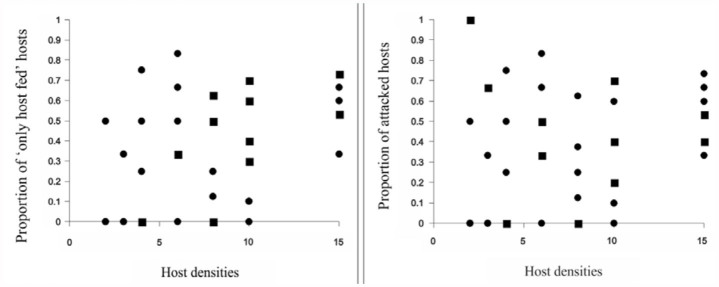
Proportion of *Tuta absoluta* larvae (a) only host-fed, and (b) attacked (host-fed and/or parasitized) by *Dineulophus phtorimaeae* in relation to host density. Type I functional responses: (a) y = 0.202 + 0.02x; (b) y = 0.233 + 0.02 x; significant at 95% confidence (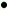 = multiple replicates; 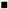 = single replicates). High quality figures are available online.

The proportions of *T. absoluta* larvae either attacked (host-fed and/or parasitized) or only host-fed were independent across the range of host densities tested, and a type-I functional response was suggested by the logistic regression because none of the parameters were significantly different from zero (Table 2, 3; Figure 4). In general, a high variability in the proportion of attacked hosts at each density was found; in particular, at intermediate hosts densities (6 to 10), the ectoparasitoid attacked about 80% of offered hosts (Figure 4a, b).

The type I model equation to estimate the instantaneous attack rate (a') fit the observed data reasonably well for attacked *T. absoluta* larvae by *D. phtorimaeae* (Table 4). In both cases, the ectoparasitoid killed approximately 20% of host larvae, independently of the host density offered.

## Discussion

The results presented here showed that *D. phtorimaeae* can be considered an extremely synovigenic (ovigeny index = 0) species, with an egg load of about 5 eggs into early female adult life (up to 5 days old). The results also showed that females gradually develop a small egg load, and they draw upon both nonhost and host food to produce eggs and start oviposition. The findings shown in Figure 2 suggest that *D. phtorimaeae* could be referred to as an anautogenous or obligate host-feeder parasitoid, that is, females oviposit only if they have previously host-fed (Jervis and Kidd 1986). In a review paper, Jervis et al. (2008) related parasitoid reproduction to patterns of nutrient allocation, utilization, and acquisition, and proposed four types of fecundity curves (types 1 to 4, from proovigenic to extremely synovigenic) supported by empirical data. Based on the information gathered in this study and the potential fecundity reported before (around 9 eggs per female (Luna et al. 2010)), the results strongly suggest that *D. phtorimaeae* exhibit a Type 4 curve that indicates “slow” traits, i.e., being extremely synovigenic, producing large, yolkrich (anhydropic) eggs, and typically having a lifetime potential fecundity of < 50 eggs. Type 4 species are likely among the least fecund in parasitic Hymenoptera. Other Type 4 primarily solitary idiobiont parasitoid species with similar reproductive traits to *D. phtorimaeae* (maximum age-specific fecundity = 10) are found among Chalcididae (*Brachymeria intermedia, B. lassus*) and Ichneumonidae (*Diapetimorpha introita*). All these species have been shown to be potential candidates for biological control under specific conditions.

Foraging behavior theory indicates that females may be forced to decide whether to feed or to oviposit (Bernstein and Jervis 2008). This forced decision, combined with egg-limitation, which implies that the number of existing hosts exceeds the number of available mature eggs, represents important constraints for maximizing reproductive success (Heimpel et al. 1998; Jervis et al. 2001). Based on population dynamics theory, Jervis et al. (1996) concluded that there is a positive relationship between destructive hostfeeding and both the establishment and success rates following introduction in classic biological control agents.

*D. phtorimaeae* females deprived of hosts, even when they were artificially supplied with a non-host diet (honey *ad libitum*), practiced egg resorption at a rate of about 1 egg per day and continued maturing eggs over their lifetime. For synovigenic parasitoids, it has been shown that carbohydrate-energetic sources cannot ensure egg production over the female's lifetime if host feeding is prevented, usually resulting in egg resorption (Jervis and Kidd 1986; Jacas et al. 2009). Host-feeding was related to sterol energetic acquisition for egg production and directly affected egg viability, as shown by Mondy et al (2006) for *Eupelmus vuilleti*. The reallocation of resources due to resorption for possible future reproductive success is an important evolutionary trait that modulates reproductive behavior. Egg resorption in the absence of hosts was also reported by Collier (1995) for *Aphytis melinus* and compared with five other parasitoid species. In the case of *A. melinus*, maximum egg load was registered at 2 days old, although this wasp emerges from pupa (day 0) with some mature oocytes. Earlier work has shown that females deprived of hosts early in life (up to 5 days old) actually attacked similar rates of hosts as females that received hosts daily (Luna et al. 2010).

The tolerance of *D. phtorimaeae* to host deprivation, in part subsidized by egg resorption, and its effect on egg load and effective parasitism are relevant pieces of information in order to deduce the realized reproductive behavior in the case of augmentative releases to control *T. absoluta*.

*D. phtorimaeae* functional response experiments indicated the same densityindependent behavior when either attacking or only host-feeding on the increasing *T. absoluta* densities. This result suggests that in conditions when a female is forced to use the host for feeding rather than parasitizing (because of an energetic-supply shortage or being a young age, for instance), *D. phtorimaeae* could inflict similar host mortality rates, although with implications on its reproductive fitness. Field mortality due to host-feeding is frequently underestimated because of the difficulty in assessing it, and despite its relevance, Jervis et al. (1996) pointed out that this biological trait should be considered as another positive criterion when selecting biological control agents.

Functional response experiments are useful in evaluating potential biocontrol candidates, but must be considered along with field-realistic patterns of parasitism as well. *T. absoluta* is attacked by a number of parasitoid species in tomato crops (Garcia Roa 1989; Miranda et al. 1998; Faria et al. 2000; Desneux et al. 2010; Luna et al. 2010, 2011; Medeiros et al. 2011). In Argentina, the koinobiont larval endoparasitoid *Pseudapanteles dignus* (Muesebeck), although presumably considered an inferior competitor, has been commonly found coexisting with *D. phtorimaeae* in the field (Luna et al. 2007, 2010, Sánchez et al. 2009). These species clearly have contrasting biologies, similar density-independent responses, and high variability in the proportion of attacked hosts to an increasing host density. In comparison, *P. dignus* has a higher attack rate and is a more time-limited parasitoid than *D. phtorimaeae*, which seems to be a more egglimited parasitoid (Heimpel et al. 1998). Parasitoids display extraordinary variation in their reproductive traits and exhibit remarkable adaptive plasticity in relation to many constraints, making them capable of coexisting in natural communities (Jervis et al. 2008).

In conclusion, the findings of this study increase the understanding of *D. phtorimaeae* reproductive biology regarding oviposition, host-feeding patterns, and functional responses. These results are critical in order to develop a *T. absoluta* biological control program, whether alone or combined in an integrated pest management scheme. Additionally, this research provides useful insights on the evolutionary importance of some of the traits displayed by *D. phtorimaeae* females. Finally, this study provides the basis to forthcoming studies on *D. phtorimaeae* non-host foods, patterns of parasitism in the field, and inter-specific competition with *P. dignus*, to advance in the knowledge of finer aspects between the two parasitoids' interaction.
